# Alpha-Synuclein Pathology Coincides With Increased Number of Early Stage Neural Progenitors in the Adult Hippocampus

**DOI:** 10.3389/fcell.2021.691560

**Published:** 2021-07-07

**Authors:** Hannah Bender, Simone A. Fietz, Franziska Richter, Milos Stanojlovic

**Affiliations:** ^1^Institute of Veterinary Anatomy, Histology and Embryology, Faculty of Veterinary Medicine, University of Leipzig, Leipzig, Germany; ^2^Department of Pharmacology, Toxicology and Pharmacy, University of Veterinary Medicine Hannover, Hanover, Germany; ^3^Center for Systems Neuroscience, Hanover, Germany

**Keywords:** Parkinson’s disease, adult neurogenesis, hippocampus, Dementia with Lewy bodies (DLB), alpha-synuclein

## Abstract

Alpha-synuclein pathology driven impairment in adult neurogenesis was proposed as a potential cause of, or at least contributor to, memory impairment observed in both patients and animal models of Parkinson’s disease (PD) and Dementia with Lewy Bodies (DLB). Mice overexpressing wild-type alpha-synuclein under the Thy-1 promoter (Thy1-aSyn, line 61) uniquely replicate early cognitive deficits together with multiple other characteristic motor and non-motor symptoms, alpha-synuclein pathology and dopamine loss. Here we report overt intracellular accumulation of phosphorylated alpha-synuclein in the hippocampus of these transgenic mice. To test whether this alters adult neurogenesis and total number of mature neurons, we employed immunohistochemistry and an unbiased stereology approach to quantify the distinct neural progenitor cells and neurons in the hippocampal granule cell layer and subgranular zone of 6 (prodromal stage) and 16-month (dopamine loss) old Thy1-aSyn mice. Surprisingly, we observed an increase in the number of early stage, i.e., Pax6 expressing, progenitors whereas the numbers of late stage, i.e., Tbr2 expressing, progenitors and neurons were not altered. Astroglia marker was increased in the hippocampus of transgenic mice, but this was not specific to the regions where adult neurogenesis takes place, arguing against a commitment of additional early stage progenitors to the astroglia lineage. Together, this uncovers a novel aspect of alpha-synuclein pathology in adult neurogenesis. Studying its mechanisms in Thy1-aSyn mice could lead to discovery of effective therapeutic interventions for cognitive dysfunction in PD and DLB.

## Introduction

Parkinson’s disease (PD) is characterized by the presence of proteinaceous cytoplasmic inclusions termed Lewy bodies (LB), with alpha-synuclein (α-syn) as a main component (Spillantini et al., 1998). Alpha-syn-associated pathology also plays a major role in neurodegenerative processes in Dementia with Lewy Bodies (DLB) and multiple system atrophy (MSA), together with PD collectively known as synucleinopathies (McCann et al., 2014). Degeneration of dopaminergic neurons in the substantia nigra pars compacta leads to the classical motor symptoms of PD (Kouli et al., 2018). In addition, non-motor symptoms including dementia, cognitive dysfunction, sleep impairments and mood disorders correlate with LB pathology in different brain regions, including olfactory bulb, medulla, pons, and various cortical regions (Braak et al., 2003; Braak and Tredici, 2011; Pfeiffer, 2016). In fact, PD and DLB combined are a leading cause of neurodegenerative dementias, second only to Alzheimer’s disease (Vann Jones and O’Brien, 2014). Importantly, early or late cognitive decline is unresponsive to current symptomatic treatments and represents a tremendous disease burden for patients (Aarsland, 2016). Thus, there is urgent need to understand the pathophysiology of cognitive symptoms in synucleinopathies. Alpha-syn-associated pathology in cortical and hippocampal brain areas is likely involved, but the precise cellular substrates and mechanisms are still elusive (Francis, 2009; Liu et al., 2019).

There are several different transgenic animal models available to investigate the effects α-syn associated pathology *in vivo*. The Thy1-aSyn mice over-express human wild-type alpha-synuclein under the control of the murine Thy-1 promoter. These transgenic mice replicate widespread α-syn pathology and are extensively studied in the context of α-syn aggregation and toxicity, mitochondrial dysfunction, neuroinflammation, and dopamine loss, all of which are integral parts of the disease. Furthermore, these mice replicate both classical motor and a plethora of non-motor symptoms characteristic for PD, rendering them as one of the most suitable models to investigate both early, prodromal and eclectic (Chesselet et al., 2012; Wang et al., 2012; Grant et al., 2014; McDowell et al., 2014; Magen et al., 2015; Cuvelier et al., 2018), as well as classical mechanisms involved in PD pathology (Rockenstein et al., 2002; Fleming et al., 2006; Chesselet et al., 2012; Watson et al., 2012; Subramaniam et al., 2014). In an effort to understand the cellular substrate of early cognitive deficits in this line of mice, we found increased levels of a marker for cell division in the hippocampal SGZ (Bonsberger et al., under review as perspective in Neural Regeneration Research). This prompted us to investigate α-syn pathology in the hippocampus, and whether adult neurogenesis is affected in Thy1-aSyn mice.

Previous studies, utilizing tissue from patients, animal and *in vitro* models, have suggested an impairment of adult neurogenesis in PD and DLB (Höglinger et al., 2004; Winner et al., 2004, 2012; Crews et al., 2008; Nuber et al., 2008; van den Berge et al., 2011; Kohl et al., 2012). In mammals, only a few brain regions retain the capacity to produce neurons throughout adult life. The two major brain regions, in which adult neural progenitor cells (NPCs) reside, are the subgranular zone (SGZ) of the hippocampus, and the subventricular zone (SVZ) of the cerebral cortex (Ming and Song, 2011; Braun and Jessberger, 2014). Non-canonical sites are reported as well and include different brain regions [reviewed by Feliciano et al. (2015)]. NPCs need to pass several consecutive developmental stages before neurons as their progeny become functionally integrated into the hippocampal circuitry. Radial glial cells (RGLs, type 1 cells) represent the early NPC subpopulation that is able to generate both glia and type 2 cells. Type 2 cells are subdivided in early transit amplifying progenitors (type 2a) and late transit amplifying progenitors (type 2b), the latter known to be committed to neuronal lineage defining the first cell of late stage NPCs subpopulation. Type 2 cells divide to generate neuroblasts (NBs, type 3 cells), which subsequently exit the cell cycle and differentiate into DG neurons (Ming and Song, 2011; Braun and Jessberger, 2014). As NPCs of the adult hippocampus proliferate, migrate and differentiate, they express stage specific cellular markers (Ming and Song, 2011; Braun and Jessberger, 2014). It is shown that newborn neurons are important for brain homeostasis in health and disease (Steiner et al., 2006; Zhao et al., 2008).

Here we present intracellular accumulation of pathological forms of α-syn in the hippocampus in Thy1-aSyn mice at an age where cognitive deficits are apparent. To decipher a potential mechanism for neuronal dysfunction, we determined the number of distinct neural NPCs and neurons, as well as astroglia in this region.

## Materials and Methods

### Animals

In this study, 6 and 16 month-old (mo) WT (WT; 6 month-old, *n* = 6, 16 month-old *n* = 6; Thy1-aSyn, 6 month-old, *n* = 6, 16 month-old *n* = 5) mice were used. Transgenic, Thy1-aSyn, mice overexpressing human wild-type α-syn under the Thy-1 promoter (Rockenstein et al., 2002) are maintained on a mixed C57BL/6-DBA/2 background as described previously (Chesselet et al., 2012). Only male transgenic mice were used along with wild-type littermates. Due to the location of the transgene on the X-chromosome, female Thy1-aSyn mice show no or subtle phenotypes in most behavioral assays (Chesselet et al., 2012). Animals were maintained on a reverse 12 h dark/light cycle, with food and water *ad libitum*. All animals were treated in accordance with the German Animal Welfare Agency (TVV31/14, T46/16) and the European guidelines (Directive 2010/63/EU).

### Immunohistochemistry

Mice were anesthetized with pentobarbital and then perfused transcardially with PBS first and then with 4% paraformaldehyde. Brains were removed, post-fixed in 4% paraformaldehyde for 24 h, cryoprotected in 10–30% sucrose in 0.1 M PBS for 3 days, frozen on dry ice and stored at −20°C. Brains were cut into coronal sections of 40 μm thickness and every 8th section from bregma −0.94 mm to bregma −4.04 mm (Paxinos and Franklin, 2004) containing the hippocampus was collected.

For cell quantification studies, sections were washed with 0.05 M PBS (3 × 10 min). Antigen retrieval for sections stained with antibodies for PCNA, Pax6, Tbr2, and Tbr1 was accomplished by heating sections in 0.01 M citrate at 96°C for 2 h. Afterward, sections were permeabilized with 1% Triton X in PBS for 40 min, treated with 0.1 M glycine solution and blocked with 5% Donkey Serum in PBS for 30 min. Gelatin buffer [0.2% Gelatin, 0.5% Triton X (pH 7.4)] was used to wash the sections (2 × 5 min). Sections were incubated in gelatin buffer containing primary antibodies at 4°C overnight. Following, sections were washed with gelatin buffer (2 × 10 min and 2 × 5 min), and incubated in gelatin buffer containing Alexa Fluor conjugated secondary antibodies and DAPI for 1 h. After another round of washing, sections were mounted on glass slides and covered with Vectashield mounting medium (Vector Laboratories, Burlingame, CA, United States).

For p-α-syn, Ki-67, nestin and GFAP analysis, sections were washed with 0.05 M TBS (3 × 10 min) and blocked using 10% NGS in TBS. Then, sections were incubated overnight in 2% NGS/0.5% Triton X in TBS with primary antibodies. Following, sections were washed with 0.05 M TBS (3 × 10 min) and incubated with secondary antibodies in 5% NGS for an hour. After another round of washing, sections were covered with ProLong Gold with DAPI mounting medium (Cell Signaling, Danvers, MA, United States).

The following primary antibodies were used: rabbit anti-Tbr1 (T-box brain protein 1) (1:100, AB10554; Merck Millipore, Burlington, MA, United States); sheep anti-Tbr2 (T-box brain protein 2) (1:100, AF61669; R&D Systems, Minneapolis, MN, United States), rabbit anti-Pax6 (paired box protein 6) (1:100, PRB-278P; BioLegend, San Diego, CA, United States); mouse anti-PCNA (proliferating cell nuclear antigen) (1:100, NB500-106; Novus Biologicals, Centennial, CO, United States); mouse anti-NeuN (hexaribonucleotide Binding Protein-3) (1:100, MAB377, Merck Millipore, Burlington, MA, United States); rabbit anti NeuN (1:500, ABN78, Merck Millipore, Burlington, MA, United States); mouse anti p-α-syn (phosphorylated alpha-synuclein (ser129) (1:500, AB51253, Abcam, United Kingdom); guinea pig anti GFAP (glial fibrillary acidic protein) (1:500, 173004, Synaptic Systems, DE); rabbit anti Ki-67 (marker of proliferation Ki-67) (1:500, MA5-14520, Thermo Fisher Scientific, United States); mouse anti nestin (1:500, #MAB353, Merck Millipore, Burlington, MA, United States). The following secondary antibodies (Thermo Fisher Scientific, United States) were used: Alexa Fluor donkey anti-mouse (488, R37114; 555, A31570); donkey anti-sheep (488, A11015); donkey anti-rabbit (555, A31572); goat anti-guinea pig (647, A21450); goat anti-mouse (488, A28175) (1:500).

### Unbiased Stereology

Unbiased stereology analysis with optical fractionator probe within the Stereo Investigator 11.1.2 software (MBF Bioscience, United States) was used to quantify the number of different cell populations in the GCL and SGZ of the hippocampus. For this, 40-μm-thick sections were used to allow for at least 25 μm dissector height within each section after dehydration and mounting. Systematic sampling of every 8th section was performed throughout the hippocampus. Sections were imaged using an Axioscope fluorescence microscope (Carl Zeiss AG, DE). Hippocampus boundaries were used to outline contours at 10× magnification. Cells were counted using a randomly positioned grid system controlled by Stereo Investigator in a previously defined region in all optical planes. Guard zones were set at 5 μm to account for damage during the staining procedure. The grid size for progenitor cell counting was set to 141 × 141 μm and counting frame was set to 100 × 100 μm. For counting of neurons, a grid size of 200 × 200 μm and a counting frame of 100 × 100 μm was used. Counting was performed on 40× magnification. Cells were counted throughout the entire GCL and SGZ of both hemispheres of each mouse to give an acceptable coefficient error, (CE, Gunderson) of 0.05 using the smoothness factor *m* = 1. CE < 0.1 is deemed acceptable within the field of stereology.

### Image Acquisition and Analysis

For NPC and neuronal IF, images were acquired using a Leica SP8 confocal laser-scanning microscope using a 40× objective. Images were acquired as single (immunofluorescence Tbr1 and NeuN) or stacks of 3 (double-immunofluorescence for PCNA and Pax6 or Tbr2) optical sections. All images were processed using Fiji [open source software (Schindelin et al., 2012)] and Adobe Photoshop CS6 software (Adobe, United States).

For p-α-syn, nestin IF and GFAP expression analysis, and Ki-67 positive cell quantification, ZEISS Axio Observer (Oberkochen, DE) was used to capture 20× images. Every 6th section from bregma −0.94 to bregma −4.04 was imaged and analyzed. For p-α-syn and GFAP expression analysis regions of interest were selected and the mean pixel intensity was measured using Fiji software (National Institutes of Health, Bethesda, MD, United States). For Ki-67 density analysis the number of Ki-67 positive cells was determined and divided by the length of the SGZ (mm) measured in Image J software (National Institutes of Health, United States). Images were processed using Adobe Photoshop CS5 (Adobe, United States). High magnification images of CA1 and DG regions (63×, oil immersion) and were obtained using ZEISS Apotome (Oberkochen, DE).

### Statistics

Data was analyzed using Prism software (GraphPad Software). PCNA + cell counts between WT and Thy1-aSyn mice were compared using two-tailed unpaired Student’s *t*-test. In order to examine the NPC subpopulation to which the difference in PCNA + cell number was attributed and to examine differences between the downstream cell populations, Pax6+/PCNA+, Tbr2+/PCNA+, Tbr1+ and NeuN + cell counts and mean GFAP and p-α-syn pixel intensity values between WT and Thy1-aSyn mice were compared using one-tailed unpaired Student’s *t*-test. *P*-values below 0.05 were considered significant.

## Results

### Phosphorylated a-Syn Accumulates in Specific Hippocampal Subregions of Thy1-aSyn Mice

**FIGURE 1:**
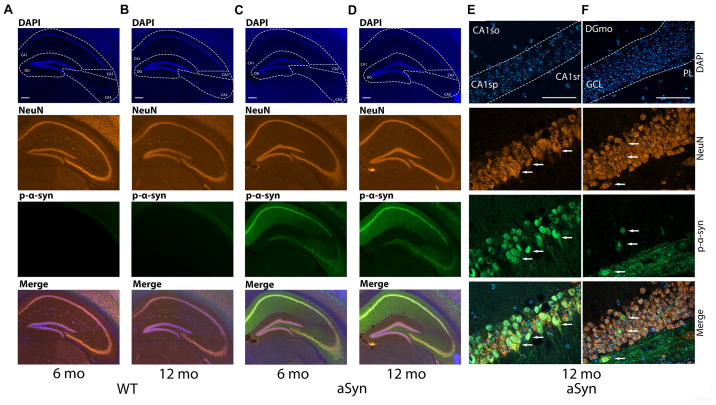
Expression of the p-α-syn in the hippocampus of the 6 and 16-month-old WT and Thy1-aSyn mice. Double-immunofluorescence for p-α-syn (green) and NeuN (orange) and DAPI staining (blue) and merged images of 6 and 16 months-old wildtype [WT; **(A,B)**], and 6 and 16 months-old Thy1-aSyn [asyn; **(C,D)**] mice. Cornu ammonis (CA1, CA2, and CA3), dentate gyrus (DG) regions. Scale bars, 200 μm. High magnification Apotome images (63×, oil immersion), double-immunofluorescence for p-α-syn (green) and NeuN (orange) and DAPI staining (blue) and merged images of Thy1-aSyn mice **(E,F)**. CA1so, CA1 stratum oriens; CA1sr, CA1 stratum radiatum; CA1sp, CA1 pyramidal layer **(E)**, GCL, DG granule cell layer; DGmo, DG molecular layer; PL, DG polymorph year **(F)**. Arrows indicate NeuN positive cells and p-α-syn accumulations. Scale bar 50 μm.

Phosphorylation of α-syn on Serin 129 (p-α-syn) is considered a toxic posttranslational modification and was found at high levels in the hippocampus using western blotting in Thy1-aSyn mice (Chesselet et al., 2012). Therefore, we first examined which subregions of the hippocampus were specifically affected by the posttranslational modification and performed immunohistochemistry for p-α-syn. We found, as expected, virtually no p-α-syn expression in WT mice in all observed hippocampal regions (CA1, CA2, CA3, and DG) in both 6 and 16 months old mice (Figures 1A,B). Interestingly, in Thy1-aSyn mice, the expression of p-α-syn is most abundant in CA1 and CA3 regions (Figures 1C,D). Although staining fills the entire cell in the CA1 pyramidal layer (CAsp), it appears that the strongest signal comes from the cell body and cell nucleus. Further, p-α-syn signal can be observed in extracellular space as well and in CA1 stratum oriens (CA1so) and stratum radiatum (CA1sr) (Figure 1E). In granule cell layer (GCL) of DG p-α-syn is expressed to a lesser extent compared to CA1sp region but in a similar fashion (Figure 1F). Signal is present in cell body and processes, being the strongest in cytoplasm and nucleus. Less p-α-syn is observed outside the cells in GCL and DG molecular layer (DGmo) in comparison to CA1 region (Figures 1E,F). Interestingly, larger quantities of p-α-syn were detected in the extracellular space of the polymorph layer (PL). Cells with p-α-syn accumulation that follow the same pattern of expression as in CA1sp and GCL can be observed in PL as well (Figure 1F).

### The Number of Early Stage NPCs, but Not Late Stage NPCs and Neurons Is Increased in the GCL and SGZ of Thy1-aSyn Mice

**FIGURE 2:**
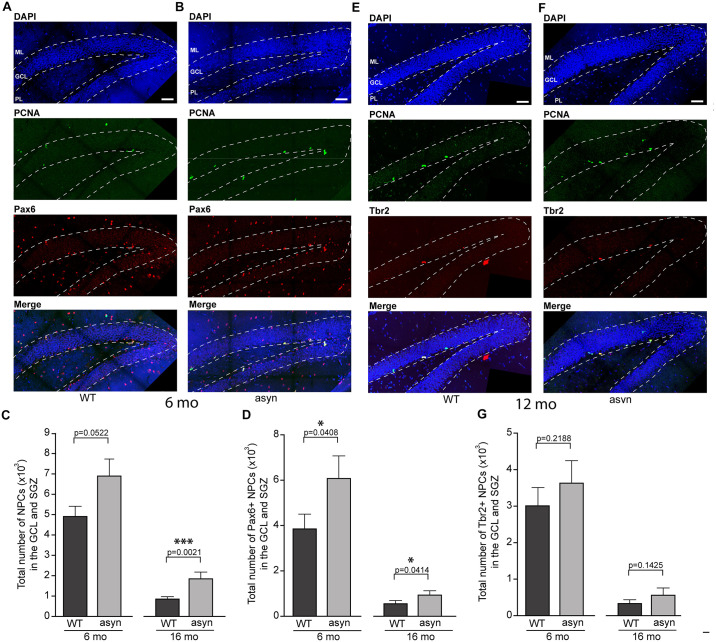
Quantification of PCNA, Pax6 and Tbr2 positive cells in the hippocampal dentate gyrus of wildtype and Thy1-aSyn mice. Double-immunofluorescence for PCNA (green) and Pax6 (red) and DAPI staining (blue) and merged images of 6-month-old wildtype [WT, **(A)**] and Thy1-aSyn [asyn, **(B)**] mice. Number of PCNA+ cells **(C)**, Pax6+/PCNA+ cells **(D)** in the granule cell layer (GCL) and subgranular zone (SGZ) of 6- and 16-month (mo)-old wildtype (WT) and Thy1-aSyn (asyn) mice. Double-immunofluorescence for PCNA (green) and Tbr2 (red) and DAPI staining (blue) and merged image of 6-month-old wildtype [WT, **(E)**] and Thy1-aSyn [asyn, **(F)**] mice. Number of Tbr2 + /PCNA + cells **(G)** in the GCL and SGZ of 6 and 16 month-old (WT) and Thy1-aSyn (asyn) mice. Data represent mean ± SEM. (WT; 6 month-old, *n* = 6, 16 month-old *n* = 6; asyn, 6 month-old, *n* = 6, 16 month-old *n* = 5; Student’s *t*-test; ^∗^*p* < 0.05, ^∗∗∗^*p* < 0.005). GCL, granule cell layer; ML, molecular layer; SGZ, subgranular zone; PL, polymorphic layer. Scale bars, 50 μm.

Interestingly, the number of total NPCs as identified by PCNA immunofluorescence was significantly elevated in 16 months old Thy1-aSyn mice compared to WT mice of the same age (Figure 2). Moreover, the number of early stage NPCs that characteristically express Pax6 was significantly increased in Thy1-aSyn mice at both 6 and 16 months of age when compared to their WT littermates (Figure 2). To further confirm this increase in early stage NPCs we determined nestin expression as well as the number of Ki-67 positive cells as a marker for cell division in the hippocampus of 6 months old Thy1-aSyn and WT mice. Significant increase in nestin expression was observed in the DG of Thy1-aSyn mice when compared to WT littermates. While nestin expression could be associated with individual neurons in WT mice, net-like pattern of expression was observed in Thy1-aSyn animals (Supplementary Figure 1). Furthermore, density of Ki-67 positive cells was significantly higher in Thy1-aSyn compared to WT mice.

**FIGURE 3:**
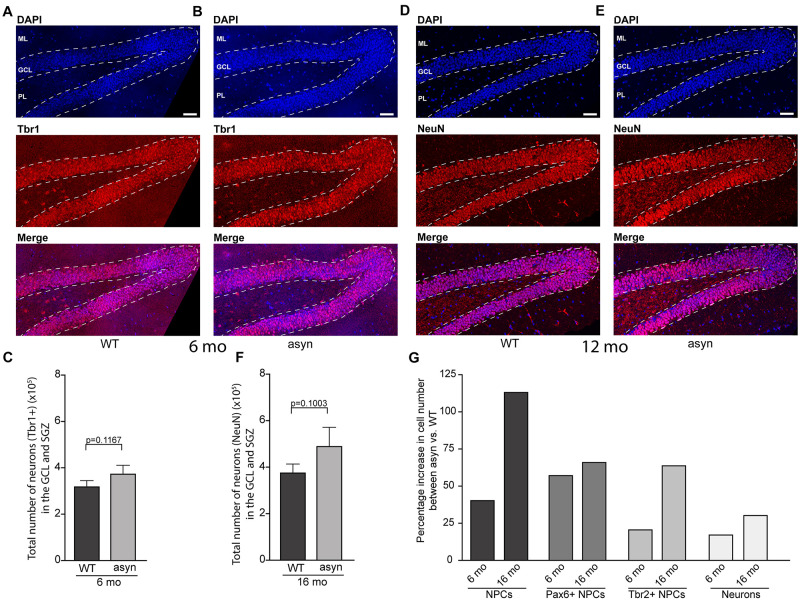
Quantification of Tbr1 and NeuN positive cells in the hippocampal dentate gyrus of wildtype and Thy1-aSyn mice. **(A,B)** Immunofluorescence for Tbr1 (red) and DAPI staining (blue) and merged images of 6-month-old wildtype [WT, **(A)**] and Thy1-aSyn [asyn, **(B)**] mice. Number of Tbr1 + cells **(C)** in the granular cell layer (GCL) and subgranular zone (SGZ) of 6-month (mo)-old wildtype (WT) and Thy1-aSyn (asyn) mice. **(D,E)** Immunofluorescence for NeuN (red) and DAPI staining (blue) and merged images of 16-month-old wildtype [WT, **(D)**] and Thy1-aSyn [asyn, **(E)**] mice. Number of NeuN + cells **(F)** in GCL and SGZ of 6 month-old wildtype (WT) and Thy1-aSyn (asyn) mice. **(G)** Change (percent) in number of PCNA+, Pax6+/PCNA+, Tbr2+/PCNA+ cells and neurons between 6 and 16 month-old Thy1-aSyn (asyn) and wildtype (WT) mice. Data represent mean ± SEM. (WT; 6 month-old, *n* = 6, 16 month-old *n* = 6; asyn, 6 month-old, *n* = 6, 16 month-old *n* = 5; Student’s *t*-test). Scale bars, 50 μm.

No significant changes were observed in the number of late stage NPCs, which characteristically express Tbr2, between WT and Thy1-aSyn mice (Figure 3). Moreover, the number of neurons as identified by Tbr1 or NeuN immunofluorescence did not differ significantly in the Thy1-aSyn mice when compared to their WT littermates at both stages analyzed (Figure 3). Together, this indicates that increased early stage NPCs do not result in more neurons in the hippocampus.

Finally, we noticed that the genotype difference appears even more pronounced in 16 months old mice, suggesting disease progression. This is demonstrated as percentage increase in numbers of NPCs and neurons between genotypes across ages (Figure 3).

### Increased Glial Fibrillary Acidic Protein (GFAP) Expression Throughout the SGZ, Molecular Layer, Polymorph Layer, and CA1 Regions of the Hippocampus

**FIGURE 4:**
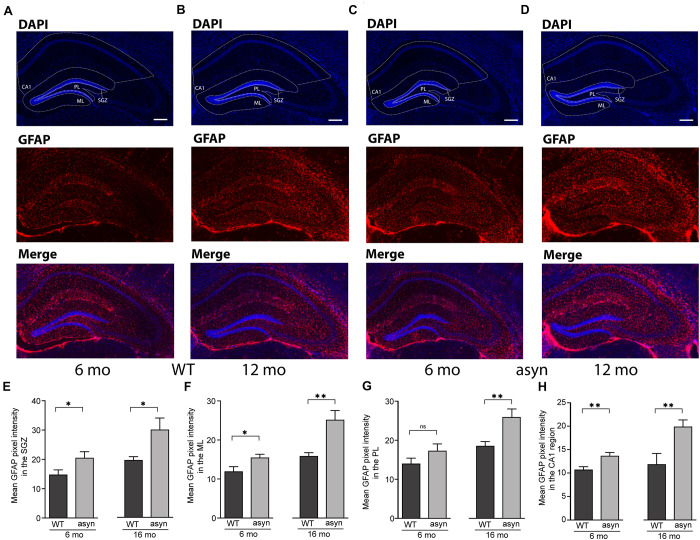
GFAP signal intensity in the hippocampal dentate gyrus and CA1 region of wildtype and Thy1-aSyn mice. **(A–D)** Immunofluorescence for GFAP (red) and DAPI staining (blue) and merged images of 6 and 16-month-old wildtype [WT, **(A,B)**] and 6 and 16-month-old Thy1-aSyn [asyn, **(C,D)**] mice. **(E–H)** Mean GFAP pixel intensity in the subgranular zone (SGZ), molecular layer (ML), polymorph layer (PL) and CA1 region of 6- and 16-month (mo)-old wildtype (WT) and Thy1-aSyn (asyn) mice. Data represent mean ± SEM. (*n* = 6/group; Student’s *t*-test; ^∗^*p* < 0.05; ^∗∗^*p* < 0.01). Scale bars, 50 μm.

Based on IF intensity measurements (Figures 4A–H), we observed an increased expression of GFAP in the SGZ (Figure 4E), molecular layer (ML) (Figure 4F), in the polymorph layer (PL) (Figure 4G), and CA1 region of 6 and 16 months old Thy1-aSyn mice compared to WT (Figures 4E–H). Observed increase in NPCs could potentially contribute to increase in astroglia, however, astrogliosis also correlates with widespread α-syn accumulation and pathology (Fellner et al., 2011). To further describe the distribution of GFAP IF across hippocampal regions, we calculated ratios between CA1 and SGZ, ML or PL, respectively, for each genotype. Comparing these rations there was no difference between WT and Thy1-aSyn mice, suggesting a general increase in reactive astrocytes throughout the hippocampus. Of note, this does not directly correlate with the apparent site and subregion specific p-α-syn pathology observed in the hippocampus (Figure 1).

## Discussion

This is the first study to report an increase in the number of early stage NPCs in the SGZ of DG of mice overexpressing α-syn (Thy1-aSyn), which apparently progresses with age. Importantly, this perturbation of adult neurogenesis does not culminate in significantly increased numbers of late stage NPCs or neurons. In addition, we observed overt intracellular accumulation of p-α-syn, a posttranslational modification thought to be neurotoxic, in the hippocampus of Thy1-aSyn mice. This demonstrates that α-syn overexpression can induce an intricate and specific alteration in the number of NPCs in the hippocampus, which may contribute to previously reported cognitive dysfunction at the examined age in these mice (Magen et al., 2015).

Studies of adult neurogenesis in postmortem tissue from PD patients are challenging and thus rare, and appear to report conflicting results. For example, a reduced numbers of proliferating cells in the SGZ and SVZ (Höglinger et al., 2004) and a decrease in the number of SOX2 (marker of early NPCs) positive cells were reported in postmortem PD brains (Winner et al., 2012). However, by using an *in vivo* and *in vitro* approach, another study proposed that adult neurogenesis is unaffected in PD patients (van den Berge et al., 2011). The decline in adult neurogenesis with age is well described and also observed in our mouse model (Figures 2, 3). By design, post mortem studies can only observe a late disease stage picture, when only few newborn neurons can be observed and thus quantified (Höglinger et al., 2004; van den Berge et al., 2011). Therefore, animal models are specifically relevant to investigate how PD related pathology may impact adult neurogenesis and thereby hippocampus related cognitive function. Previous studies observed significantly less total NPCs and young neurons in the olfactory bulb and in the SGZ of mice with overexpression of human WT or a rare form of mutated α-syn under control of the platelet-derived growth factor-β (PDGF) promoter (Winner et al., 2004; Crews et al., 2008). In mice with conditional expression of α-syn, a decreased survival of newly generated cells was observed with no change in the total numbers of NPCs (Nuber et al., 2008). Interestingly, in mice overexpressing mutated α-syn, fluoxetine ameliorates adult neurogenesis impairment via induction of neurotrophic factors expression (Kohl et al., 2012). Importantly, a recent study suggest that intracellular A53T mutant α-syn impairs adult hippocampal neuronal integration (Regensburger et al., 2020). Intriguingly, our results present an increase in the number of early but not late stage NPCs in the hippocampal GCL and SGZ of Thy1-aSyn mice, coinciding with accumulation of p-α-syn in the hippocampus and cognitive dysfunction. Controversial results from different models could for example be related to expression of WT versus mutated α-syn and region specific expression levels determined by the choice of promoter and the insertion site of the transgene among other factors. In Thy1-aSyn mice, previous western blotting demonstrated moderate expression of human WT α-syn in the DG, with higher levels of expression in the CA regions, thus reflecting physiological expression of the protein and previously found region specific vulnerability to α-syn toxicity (Chesselet et al., 2012). Mutated protein with its proposed higher toxicity may be more likely to induce cell autonomous effects if expressed by DG neurons of patients with these rare mutations (Regensburger et al., 2020). Interestingly, p-α-syn which is associated with toxicity and considered as one of the drivers of PD and DLB associated pathology (Samuel et al., 2016) was shown to accumulate in the hippocampus of Thy1-aSyn mice by western blotting (Chesselet et al., 2012). Here we show that p-α-syn is present in high abundance in CA regions but only moderately accumulates in the DG of Thy1-aSyn mice. This may explain why we did not observe alterations in numbers of adult neurons in the DG, and why early cognitive deficits in this model are moderate, rather replicating early dementia in idiopathic PD (Magen et al., 2015).

What is the destiny of the additional early NPCs generated if not the majority of them transit to late stage, hence neuronal, NPCs? GFAP expression was not specifically increased in the DG compared to CA1 in Thy1-aSyn, but it might be possible that number of additional astrocytes generated by NPCs is simply too small to be detected in our analysis. Moreover, as the genotype differences appear to progress with age, it might be speculated that hippocampal neurogenesis is altered at far advanced stages of the disease, i.e., in Thy1-aSyn mice older than 16 month-old of age. Studies to further characterize the fate of the early NPC downstream cell populations, and functional implications, are underway.

To explain the mechanism that underlie the observed increase in numbers of early stage NPCs and their potential destiny, we propose three scenarios. First, Thy1-aSyn mice show an increase in extracellular dopamine levels at 6 months of age, and a loss of dopamine at 14 months of age (Chesselet et al., 2012). Dopamine, through dopamine receptors, plays an important role in NPCs proliferation, migration, and differentiation (Borta and Höglinger, 2007; Berg et al., 2013) and induces an increase in the number of NPCs in animal models of PD (Höglinger et al., 2004; O’Keeffe et al., 2009; Winner et al., 2009). Another possible explanation could be the loss of inhibitory input, circuitry remodeling, and hippocampal network hyperexcitability which is proposed as present in DLB pathophysiology (Morris et al., 2015), and has been observed in Thy1-aSyn as well as in mice overexpressing rare α-syn mutations (Morris et al., 2015; Singh et al., 2019). As documented for epileptic seizures, such alterations of neuronal activity may promote adult neurogenesis (Jessberger and Parent, 2015). Finally, α-syn pathology through gain or loss of function may directly impact mechanisms of cell cycle regulation along the process of neurogenesis, leading to NPCs stuck or shifting in proliferation or maturation processes (Lee et al., 2003; Winner et al., 2012; Rodríguez-Losada et al., 2020; Findeiss et al., 2021). If α-syn pathology spreads across synapses (Chu and Kordower, 2015; Melki, 2018), none of the above mechanisms require a direct intracellular toxicity in DG neurons, which could be argued against given the relatively low expression level of α-syn in this region, physiologically and in Thy1-aSyn mice as discussed above.

Further studies are needed in order to draw solid conclusions on the mechanisms of how α-syn overexpression specifically increases early stage NPC numbers, and the impact on its downstream cell populations and hippocampal function. Understanding the role of adult neurogenesis, and more generally the hippocampus, in cognitive deficits not amenable to current treatment options in PD and DLB represents an urgent need. Thy1-aSyn mice could provide a unique tool to test the above discussed potential mechanisms, and to assess targeted therapeutic interventions with cognitive dysfunction as functional endpoint.

## Data Availability Statement

The raw data supporting the conclusions of this article will be made available by the authors, without undue reservation.

## Ethics Statement

The animal study was reviewed and approved by the Saxonian German Animal Welfare Agency (TVV31/14, T46/16).

## Author Contributions

HB, SF, and FR: conceptualization of the study. HB: implementation of research, analysis, and interpretation of data. HB, MS, SF, and FR: conduction of the experiments, analysis, and interpretation of data. MS: the manuscript initial draft. All authors: the manuscript critical correction and approval of final version.

## Conflict of Interest

The authors declare that the research was conducted in the absence of any commercial or financial relationships that could be construed as a potential conflict of interest.

## Supplementary Material

Supplementary Figure 1 Nestin expression and Ki-67 positive cell density in the hippocampal dentate gyrus of 6 months old WT and Thy1-aSyn mice. Double-immunofluorescence for nestin (orange), Ki-67 (green), DAPI staining (blue) and merged apotome images of wildtype [WT: 20×, **(A)**; 63× oil, **(B)**], and Thy1-aSyn [aSyn; 20×, **(C)**; 63× oil, **(D)**] mice. DG, dentate gyrus; GCL, granule cell layer; DGmo, DG molecular layer; PL, DG polymorph year **(A1–D1)**. Arrows indicate co-localized Ki-67 positive cells and nestin puncta. **(E)** Mean nestin pixel intensity in the DG of 6-month (mo)-old wildtype (WT) and Thy1-aSyn (asyn) mice. **(F)** Density of Ki-67 positive cells in the subgranular zone (SGZ) of 6-month (mo)-old wildtype (WT) and Thy1-aSyn (aSyn) mice. Data represent mean ± SEM. (*n* = 6/group; Student’s *t*-test; ^∗^*p* < 0.05; ^∗∗^*p* < 0.01). Scale bars: 200 μm for 20× images **(A1,C1)**; 50 μm for 63× images **(B1,D1)**.
