# Supplementary figures and images for: Alpha-Synuclein Pathology Coincides With Increased Number of Early Stage Neural Progenitors in the Adult Hippocampus
Source: Front Cell Dev Biol. 2021 Jul 7;9:691560. doi: 10.3389/fcell.2021.691560 (PMC8293917; doi:10.3389/fcell.2021.691560)

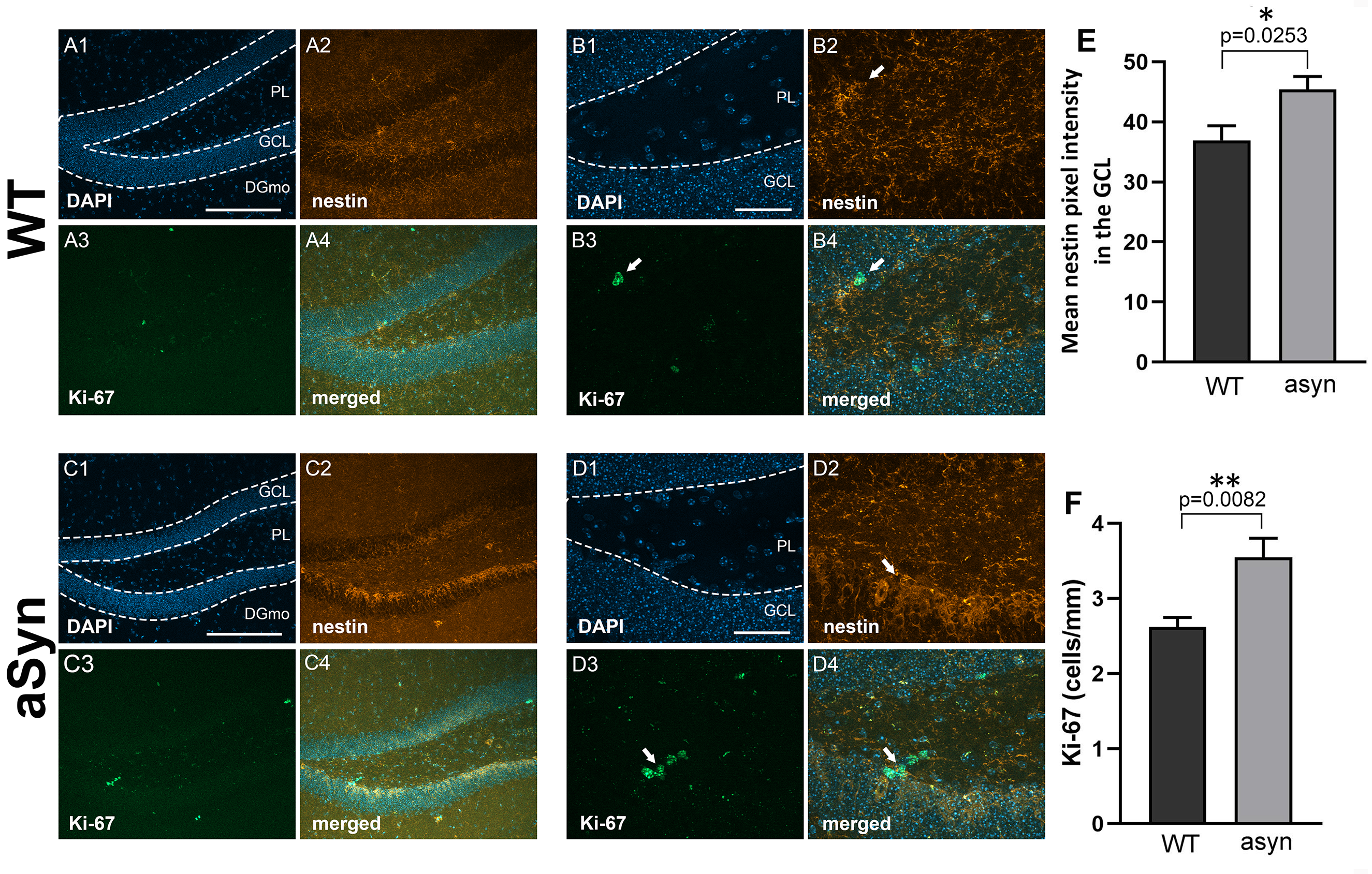

Supplement: Supplementary Figure 1 — Nestin expression and Ki-67 positive cell density in the hippocampal dentate gyrus of 6 months old WT and Thy1-aSyn mice. Double-immunofluorescence for nestin (orange), Ki-67 (green), DAPI staining (blue) and merged apotome images of wildtype [WT: 20×, (A); 63× oil, (B)], and Thy1-aSyn [aSyn; 20×, (C); 63× oil, (D)] mice. DG, dentate gyrus; GCL, granule cell layer; DGmo, DG molecular layer; PL, DG polymorph year (A1–D1). Arrows indicate co-localized Ki-67 positive cells and nestin puncta. (E) Mean nestin pixel intensity in the DG of 6-month (mo)-old wildtype (WT) and Thy1-aSyn (asyn) mice. (F) Density of Ki-67 positive cells in the subgranular zone (SGZ) of 6-month (mo)-old wildtype (WT) and Thy1-aSyn (aSyn) mice. Data represent mean ± SEM. (n = 6/group; Student’s t-test; ∗p < 0.05; ∗∗p < 0.01). Scale bars: 200 μm for 20× images (A1,C1); 50 μm for 63× images (B1,D1). [file Image_1.tif]
